# National Health and Modern Problems

**Published:** 1920-07-24

**Authors:** 


### National Health and Modern Problems.

*To the Editor of* The Hospital.

Sir,—Your correspondent, "Contemplator," takes up a partly Ruskinian attitude. But that is vain. Mankind's will to power, and the progress from homogeneity to heterogeneity, which, as was long ago pointed out, is inherent in the nature of things, will always make life a continually increasing complexity. No doubt from some points of view it would be better if life could be simplified. It is on record that some Danish missionaries, sent to convert a certain uncivilised tribe, very honestly went home again, averring truly that these people were perfectly happy and well conducted, and in no need of any teaching, ethically, from Europeans. Again, if some new source of power became available, and—much bigger supposition!—were not monopolised by anyone, we might be in the position of those Polynesians who, living by the breadfruit, are spared economic anxiety. This last instance is not a very good one, because in science and art we have plenty of field for endeavour, even were our living sure. But still both cases strike one as not perfectly desirable. There is not much of the "aspiring will of humanity to divinity" about them, not much of man's unconquerable spirit. No! the remedy is to accept industrialism, but to divorce it from squalor.

In this country we have not reached the first steps of that process. It must be ten years ago now that Sheffield, for instance, sent a commission of inquiry to report on Sölingen. That commission duly reported the specially-made briquettes which burnt smokelessly in the factories, so that conifers could grow in the district, and the "workshop factories" attached to model dwellings, so that many men saved the time journeying to and fro from their job, and worked more as artists than as "hands." But of course nothing was done, and Sheffield is just as bad as ever (unfortunately I was over there the other week). Again, other travellers have told us of the pit-head baths at coalmines in Germany and Belgium, how the colliers went to work dressed like anyone else, unlocked their lockers, changed into working things, went down, worked, came up again, bathed, changed back and returned home not looking like "underground savages" (an English collier's description of his own appearance). Look now at Ashington, or Mexborough or Cannock Chase!

However, all this is the A B C. The rest of the subject is a political question—more than that, an international one. I think myself that events are in train. But you, Sir, and "Contemplator" as well, will excuse me from treading upon such delicate ground.—I am yours, etc.,

Caduceus.

